# Orthoflaviviral Inhibitors in Clinical Trials, Preclinical In Vivo Efficacy Targeting NS2B-NS3 and Cellular Antiviral Activity via Competitive Protease Inhibition

**DOI:** 10.3390/molecules29174047

**Published:** 2024-08-27

**Authors:** Lorenzo Cavina, Mathijs J. Bouma, Daniel Gironés, Martin C. Feiters

**Affiliations:** 1 Institute for Molecules and Materials, Faculty of Science, Radboud University, Heyendaalseweg 135, 6525 AJ Nijmegen, The Netherlands; boumamathijs@gmail.com (M.J.B.); daniel@girones-pbcs.com (D.G.); 2 Protinhi Therapeutics, Transistorweg 5, 6534 AT Nijmegen, The Netherlands

**Keywords:** orthoflavivirus, dengue virus, zika virus, West Nile virus, protease, NS2B-NS3 inhibitor, in vivo efficacy

## Abstract

Orthoflaviviruses, including zika (ZIKV), West Nile (WNV), and dengue (DENV) virus, induce severely debilitating infections and contribute significantly to the global disease burden, yet no clinically approved antiviral treatments exist. This review offers a comprehensive analysis of small-molecule drug development targeting orthoflaviviral infections, with a focus on NS2B-NS3 inhibition. We systematically examined clinical trials, preclinical efficacy studies, and modes of action for various viral replication inhibitors, emphasizing allosteric and orthosteric drugs inhibiting NS2B-NS3 protease with in vivo efficacy and in vitro-tested competitive NS2B-NS3 inhibitors with cellular efficacy. Our findings revealed that several compounds with in vivo preclinical efficacy failed to show clinical antiviral efficacy. NS3-NS4B inhibitors, such as **JNJ-64281802** and **EYU688**, show promise, recently entering clinical trials, underscoring the importance of developing novel viral replication inhibitors targeting viral machinery. To date, the only NS2B-NS3 inhibitor that has undergone clinical trials is **doxycycline**, however, its mechanism of action and clinical efficacy as viral growth inhibitor require additional investigation. **SYC-1307**, an allosteric inhibitor, exhibits high in vivo efficacy, while **temoporfin** and **methylene blue** represent promising orthosteric non-competitive inhibitors. **Compound 71**, a competitive NS2B-NS3 inhibitor, emerges as a leading preclinical candidate due to its high cellular antiviral efficacy, minimal cytotoxicity, and favorable in vitro pharmacokinetic parameters. Challenges remain in developing competitive NS2B-NS3 inhibitors, including appropriate biochemical inhibition assays as well as the selectivity and conformational flexibility of the protease, complicating effective antiviral treatment design.

## 1. Introduction

The *Orthoflavivirus* (formerly known as *Flavivirus*) genus comprises more than 50 positive-sense and single-stranded RNA viruses [1,2] and belongs to the *Flaviviridae* family [3]. Zika virus (ZIKV, *O. zikaense*), West Nile virus (WNV, *O. nilense*), and the four serotypes of dengue virus (DENV1-4, *O. denguei*), all transmitted by mosquitoes, are the most prevalent and clinically relevant members of the genus. Infections with ZIKV and WNV can lead to neurological syndromes such as encephalitis, whereas both ZIKV and DENV infections may result in visceral diseases like hemorrhagic syndromes [1,2]. DENV alone accounts for an estimated 390 million infections annually, with nearly 100 million of these cases being clinically manifested and a significant majority occurring in Asia (70%) [4]. WNV has caused 7 million infections in the United States within a 20-year period, with 51,000 clinical cases and 2300 reported fatalities [5]. Although ZIKV typically causes mild symptoms in its 850,000 recorded cases, it can lead to fetal deformities if transmitted during pregnancy, with 3700 cases of congenital birth defects reported in the Americas before 2018, especially throughout a major outbreak that occurred in 2015–2016 [6].

Despite the global impact of these viral infections, no clinically approved antiviral treatments are currently available. The high infection rates and severe health consequences of orthoflavivirus infections underscore the importance of developing effective therapeutics [1,2,4,5,6]. Research efforts have focused on the structural and biochemical properties of orthoflaviviruses, leading to the development of vaccines for Japanese encephalitis virus (JEV), yellow fever virus (YFV), tick-borne encephalitis virus (TBEV), and DENV [7,8,9,10]. There are as yet no approved vaccines against ZIKV and WNV [11,12], and DENV vaccine development remains challenging due to the existence of four distinct serotypes, which can cause antibody-dependent enhancement (ADE) upon subsequent infections with different serotypes [10,13]. Two approved vaccines, Dengvaxia and Qdenga, are available for preventing severe dengue infections. After the fatalities of numerous children in the Philippines due to ADE following vaccination with Dengvaxia [14], Sanofi modified the application of its vaccine [15] for individuals with documented prior dengue infection and who are living in endemic areas [16,17]. Additionally, in 2023 Takeda withdrew the application for Qdenga to be approved in the U.S. [18,19]. DENV presents high genetic variability also among different strains of the same serotype, complicating further the development of an effective antiviral treatment [10]. Overall, the progress of formulating vaccines, pharmaceuticals, and immunotherapies for the prevention or treatment of dengue has been hindered by the intricate interplay between the dengue vector, virus, host, and disease pathogenesis [10,20].

**Figure 1:**
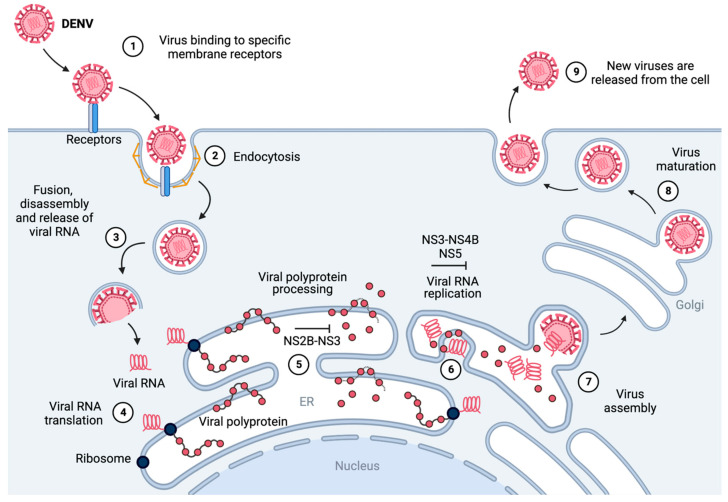
Schematic representation of the replication cycle of dengue virus [31,40]. (1) The virus binds to host-cell membrane receptors and (2) enters the host cell via clathrin-mediated endocytosis. (3) After internalization, the viral particle disassembles and fuses with the endosome, releasing the viral RNA into the cytoplasm. (4) The viral RNA is translated at the rough endoplasmic reticulum into a viral polyprotein. (5) The viral polyprotein is processed by host and viral proteases (such as NS2B-NS3), releasing seven non-structural (NS1, NS2A, NS2B, NS3, NS4A, NS4B, and NS5) and three structural proteins (C, prM, and E). (6) The NS proteins (such as NS3-NS4B and NS5) and the ER (endoplasmic reticulum) form a convolution called the replication complex, which replicates the viral RNA [31]. (7) The replicated genome and translated viral structural proteins are assembled, and immature virus particles stem from the surface of the ER. (8) Viral particles undergo trafficking via the trans-Golgi network, maturing into their infectious conformation. (9) The fully developed virions are liberated from the host cell, thereby facilitating the propagation of infection to neighboring cells. Figure generated with BioRender.com.

In addition to vaccine development, therapeutic strategies targeting viral replication have garnered interest. Among these, small molecules targeting various components of the DENV replication machinery have demonstrated potential as antiviral agents. An overview of the DENV life cycle (those of ZIKV and WNV are similar) highlighting therapeutic targets is shown in Figure 1. A prime target for such inhibitors is the NS2B-NS3 protease, a complex responsible for the cleavage of the viral polyprotein, which is essential for viral replication [21]. Several small-molecule inhibitors have been designed to specifically target the active site of this protease, thereby disrupting its catalytic activity and impeding the viral life cycle [22]. Furthermore, the NS2B-NS3 protease is responsible for cleavage of host proteins in infected cells [23,24]. Additionally, NS2B is known to help evade the antiviral innate immune response by interacting with the host-immunomodulating proteins MAVS and IKKε [25]. Similarly, NS3 is reported to modulate host chaperone proteins, silencing the innate immune antiviral response [26]. NS3 also has an RNA helicase domain, which ensures efficient translation of the viral genome [27]. The NS3 helicase domain requires acetylation by host acetyltransferase KAT5γ to exert its activity, representing a drug target distinct from the viral protease [28]. The NS2B-NS3 protease is largely conserved across the four DENV serotypes as well as WNV and ZIKV [29], making it an attractive target for the development of anti-orthoflaviviral inhibitors [30]. Another promising target is the NS3–NS4B interaction, which plays a crucial role in facilitating the viral replication complex assembly [31,32]. Inhibitors targeting this interface have shown potential in disrupting the formation of the replication complex, thus hindering viral RNA synthesis [33]. Another relevant target is NS5, which acts as a viral RNA polymerase (RNA-dependent RNA polymerase, RdRp) [34] and methyltransferase [35] in the replication complex [31] but also migrates in the host cell nucleus to modulate the host’s antiviral response [36]; the developments in NS5-targeting compounds have recently been reviewed [37]. Other viral and host machineries have been successfully targeted to combat orthoflaviviral infections in a preclinical setting, but as none of those have yet yielded a clinical candidate, they are not covered in this work; we refer to reviews on the topic collecting the most recent developments [38,39]. In this review, we look at orthoflaviviral replication inhibitors (i.e., small-molecule compounds exerting a virucide effect, thus directly blocking viral replication) that have entered clinical trials. Compounds modulating the immune response or acting on the symptomatic form of the disease, as well as biologics, have been excluded. Additionally, we review NS2B-NS3 protease inhibitors with reported in vivo efficacy. Lastly, we focus on competitive broad-spectrum anti-orthoflaviviral NS2B-NS3 inhibitors with reported cellular antiviral activity in vitro under 10 µM.

## 2. NS2B-NS3 Functional Analysis

**Figure 2:**
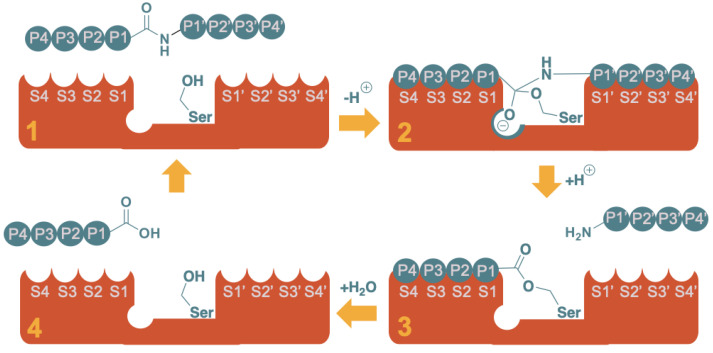
Schematic representation of the proteolysis of an octapeptide by a serine protease. The mechanism can be rationalized in four major stages: (1) Substrate recognition; (2) Nucleophilic attack and formation of the tetrahedral intermediate (note the oxyanion hole stabilizing the negative charge); (3) Release of the “prime” fragment (H-P1’-P2’-P3’-P4’) as a free amine and formation of the acyl protease intermediate with the non-prime fragment (P4-P3-P2-P1-O-[Ser]); (4) Regeneration of the active site, releasing the non-prime fragment as free carboxylic acid.

The activity of NS2B-NS3 is schematically represented in Figure 2, where eight subsites (from left to right: S4, S3, S2, S1, S1’, S2’, S3’, S4’) are identified in the protease, which recognize as many amino acids (P4, P3, P2, P1, P1’, P2’, P3’, P4’) from the targeted peptide sequence to be cleaved [41,42]. Most relevant for binding to the protease are the S1–S4 subsites, which in the case of NS2B-NS3, accommodate a sequence of cationic amino acids, such as Lys and Arg. Hydrolysis of the amide bond takes place between the so-called P1 and P1’ residues by a nucleophilic serine present at the center of the catalytic site and part of a catalytic triad (Asp-His-Ser). The Asp-His pair deprotonates the side chain -OH of the catalytic serine, increasing its nucleophilicity. As shown in Figure 2, the catalytic serine attacks the carbonyl of the P1 residue, forming a tetrahedral intermediate, whose oxyanion moiety is thought to be stabilized by the so-called oxyanion hole [42]. Collapse of this intermediate results in the release of the P1’ to P4’ peptide fragment and yields an acyl protease intermediate with the P1 to P4 remaining peptide. The active site is then regenerated via hydrolysis of the ester by an adventitious molecule of water and positioned by an intricate web of hydrogen bonds from the protease, releasing the P1–P4 fragment.

## 3. Mechanisms of Enzyme Inhibition

In the exploration of the structural design and efficacy of inhibitors of viral proteases, kinetic and structural analyses play crucial but distinct roles in understanding their mechanism of inhibition and influence the different stages and strategies in their development as new antiviral therapeutics. In the kinetic analysis, which focuses on how a drug inhibits its target enzyme, including the nature and strength of the inhibition, competitive, non-competitive, and uncompetitive inhibition are distinguished. The activity of a competitive inhibitor depends on the concentration of both the inhibitor and the substrate, while that of a non-competitive inhibitor will not be affected by the substrate concentration. In uncompetitive inhibition, the inhibitor binds only to the enzyme–substrate complex and not to the free enzyme; this form of inhibition is unique in that it requires the enzyme to first bind to its substrate before the inhibitor can bind [43]. In the structural analysis of inhibition, the molecular architecture of the target and its binding interactions with the drug are considered, and binding to the orthosteric site, the active site, is distinguished from binding to an allosteric site [44], a different site that modulates the activity of the enzyme. 

The most straightforward type of inhibition is competitive orthosteric, where the inhibitor competes directly with the substrate for the active site [43]. However, in some cases orthosteric enzyme inhibition can also result in non-competitive behavior, for instance in cases where compounds bind to the active site but induce conformational changes affecting the affinity for the natural substrate or the catalytic activity [45,46]. Herein, we refer to such compounds as non-competitive orthosteric inhibitors. It is important to note that this kind of inhibition is distinct from uncompetitive inhibition, a mechanism through which none of the NS2B-NS3 protease inhibitors discussed here were reported to act.

Parallelly, the general kinetic of allosteric inhibition is non-competitive, where the inhibitor binds to a site distinct from the active site, leading to structural changes that reduce enzyme activity without direct competition with the natural substrate [47,48]. However, allosteric inhibition can also show competitive behavior, for instance when binding of the substrate affects the structure of the allosteric site [49,50]. In addition, an inhibitor may bind covalently to the active site, either reversibly or irreversibly. Irreversible competitive covalent inhibitors initially compete with the substrate for binding to the active site. Upon binding, they form a covalent bond with the enzyme, leading to irreversible inactivation. Reversible competitive covalent inhibitors also form a covalent bond with the enzyme, but this bond can be cleaved (often hydrolyzed), re-forming the free enzyme and inhibitor [51].

## 4. NS2B-NS3 Protease Structural Analysis

**Figure 3:**
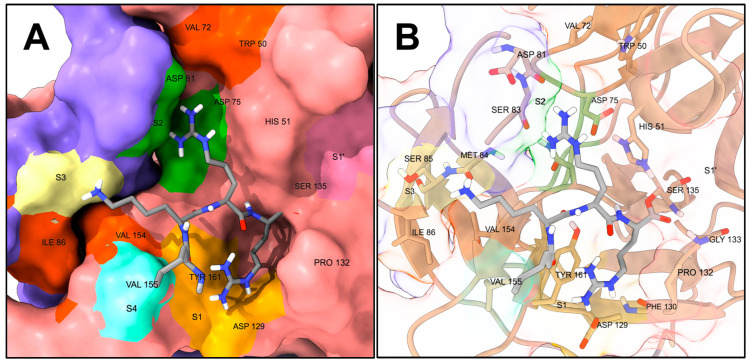
Snapshot of DENV2 NS2B-NS3 bound to the peptide aldehyde Bz-nKRR-H (PDB: 3D1I) [31]. Benzoyl-Nle-Lys-Arg-Arg-H (**Bz-nKRR-H**) is in dark gray. Only non-polar hydrogens are shown; the Bz- fragment was hidden for displaying purposes. (**A**) Representation showing the solvent-excluded surface of the protease functionally colored: the light pink surface is from NS3 and the violet from NS2B; S1 is highlighted in light orange, S2 in green, S3 in light yellow, S4 in aquamarine, and S1’ in dark pink; hydrophobic surfaces (generally not addressed as specificity subsites per se) are highlighted in dark orange. (**B**) Representation showing only the side chains and backbones of relevant amino acids of NS2B-NS3 as sticks; the protease is colored in tan and the rest of its backbone is represented as ribbon. Figure generated with UCSF ChimeraX v1.4 [54,55].

The crystal structure of the DENV3 NS2B-NS3 bound to the peptide-like inhibitor **Bz-nKRR-H** (Benzoyl-Nle-Lys-Arg-Arg-H) was solved in 2012 (PDB: 3D1I) [52]. More recently, the conformation and assembly of the DENV NS2B-NS3 was studied using NMR and molecular dynamics [53]. It is a serine protease, composed of the co-activator NS2B and the essential subunit NS3. The catalytic triad of amino acids Ser135-His51-Asp75 responsible for the cleavage of the P1-P1’ amide bond is located in the NS3 subunit, while the NS2B co-activator contributes to the formation of the S2 and S3 subsites [31,34]. The specificity and location of the S1-S4 subsites is shown for the crystal structure of DENV3 NS2B-NS3 in Figure 3, and it was determined by the positioning of the P1-P4 residues of the **Bz-nKRR-H** ligand crystallized within. 

Most of the binding region of the protease is shallow; however, the S1 subsite is generally considered the best-defined, and it is characterized by the side chain of Tyr161 and the side chain of Asp129, which respectively display cation–pi interaction and form an ion pair with the guanidinium of the P1 Arg of **Bz-nKRR-H** [52,56]. Additionally, Pro132 encases the side chain of P1, creating an open cavity defining the S1 subsite. The oxyanion hole, located in the vicinity of the catalytic Ser135 and characterized by the backbone N-Hs of Ser135 and Gly133, also represents an appealing druggable volume, being more buried and furnishing a cavity rich in polar interactions [29,52,56]. The S2, S3, and S4 subsites of the protease are more exposed to the solvent than the S1 or the oxyanion hole, and apart from the presence of Asp89 between S2 and S3 and the catalytic triad Asp75-His51-Ser135 between S1 and S2, do not present significantly buried binding surfaces. However, as explained in the literature [52,56,57,58], there is a hydrophobic area between the S3 and S4 subsites created by the interface of NS2B-NS3 (Ile86 and Val154, dark orange surface in Figure 3), which can potentially be targeted by hydrophobic substituents [59,60,61,62]. Similarly, beyond the S2 subsite, away from the catalytic center, the Trp152 and Val72 side chains make for an additional hydrophobic surface. Many orthosteric inhibitors showing non-competitive behavior are present in the literature, and such compounds are likely to bind to some parts of the S1–S4 subsites. 

It is important to note that the orthoflaviviral NS2B-NS3 complexes commonly employed for evaluating biochemical protease inhibition and for determining crystal structures are typically fusion constructs in which NS2B and NS3 are covalently linked by a polyglycine chain [52,56,63,64].

## 5. NS2B-NS3 Inhibition

**Figure 4:**
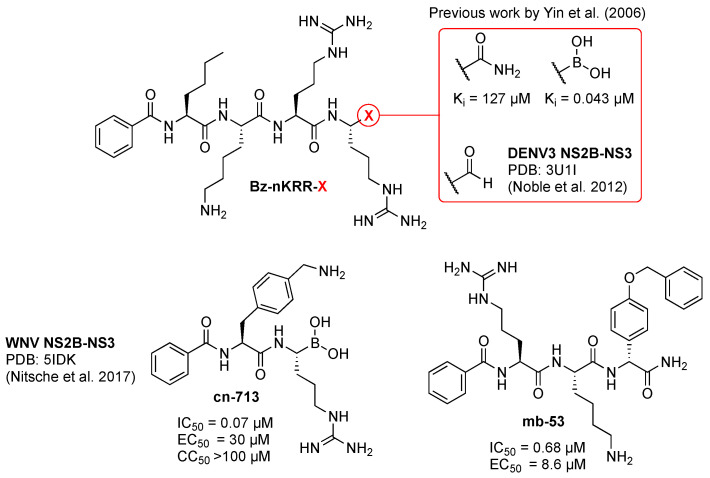
Previously reported orthoflaviviral protease peptide-like inhibitors. K_i_ and IC_50_ values are relative to DENV2 NS2B-NS3 inhibition and EC_50_ to efficacy of DENV2-infected cells. Data for **Bz-nKRR-X** are reported in references [52,68,69], for **cn-716** in reference [63], and for compound **mb-53** in reference [72].

A successful strategy to produce effective viral protease inhibitors is to generate a mimic of the P1–P4 peptide fragment, which would compete with the natural substrate for the S1–S4 subsites, impeding further cleavage of the viral polyprotein [65]. Additionally, an electrophilic trap (viz., warhead) reacting with the nucleophilic catalytic serine of NS2B-NS3 can be included at the C-terminus of the P1-mimic, generating an inhibitor that will bind covalently to the active site. Covalent viral protease inhibitors have already been successfully marketed, e.g., Narlaprevir, Arlansa^®^ against HCV (hepatitis C virus, from another genus of the *Flaviviridae*) [66] and Nirmatrelvir, Paxlovid^®^ against SARS-CoV-2 (severe acute respiratory syndrome coronavirus 2) [67]. This strategy has been exploited before [68,69], improving the inhibition of DENV NS2B-NS3 by the peptide **Bz-nKRR-NH_2_** 3000-fold, by substituting the C-terminal amide with a boronic acid warhead (Figure 4). Formation of the protease-inhibitor covalent complex locks the enzyme in an inactive state amenable to crystallographic studies. For instance, the C-terminal aldehyde (**Bz-nKRR-H**) made it possible to solve the bound crystal structure of DENV3 NS2B-NS3 (Figure 3) [52], and peptide-hybrid C-terminal boronic acid **cn-716** that of WNV [63] and ZIKV [70]. From the previously reported SAR studies of covalent orthoflaviviral protease inhibitors, it emerged that covalent inhibition requires the presence of additional, specific molecular recognition elements [63,71] and that it is possible to achieve comparable inhibition optimizing the peptide sequence without introducing an electrophilic warhead (**mb-53** in Figure 4) [63,72]. The inhibition of viral proteases has previously proven effective in treating various viral diseases; nevertheless, NS2B-NS3 inhibitors remain underrepresented in the realm of clinical development. Biochemical optimization for orthoflaviviral protease inhibitory activity of peptide-like inhibitors has often not translated into improvement of antiviral cellular efficacy, hampering the development of effective competitive inhibitors [59]. This has been ascribed principally to both the artificial nature of the fusion protein construct mostly used to assess biochemical viral protease inhibition and the cellular environment, requiring membrane permeability and proteolytic stability features, which are difficult to optimize on small cationic peptides [59]. Extensive work has been performed to improve the protease biochemical assays, both in terms of the type of NS2B-NS3 construct and assay conditions. It has been established that the unlinked NS2B-NS3 construct, as opposed to the linked or self-cleavable constructs, exerts better enzymatic activity [73]. Unfortunately, and despite being closer to the natural form, the unlinked protease is still not the primary choice for most researchers in the drug discovery field, mainly due to the associated time-consuming efforts and extra costs. Recently, it has been reported [74] that variations in assay conditions, viz. pH, salinity, buffers, and temperature, can modify the binding affinity of inhibitors as well as the proteolytic capacity. This shows the importance of choosing the right conditions, particularly in the validation of new hits.

In addition to competitive inhibition, many non-competitive NS2B-NS3 inhibitors are reported as orthosteric (reviewed in the later sections) in the literature, inhibiting the protease by binding to parts of the active site in NS3 and disrupting the functional interactions with the NS2B protein domain. These inhibitors could be classified more accurately as protein–protein interaction (PPI) inhibitors.

**Figure 5:**
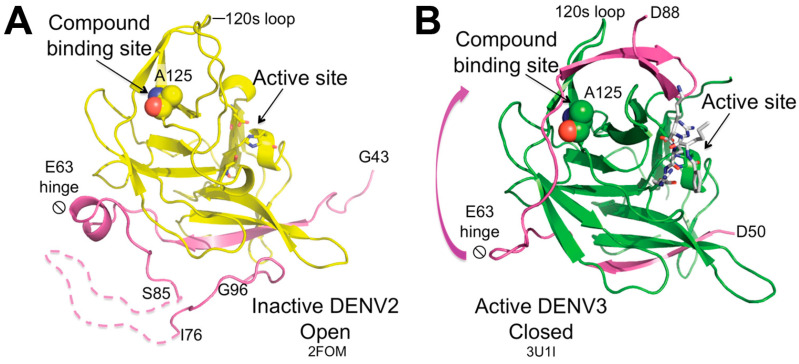
Crystal structures of NS2B-NS3 in (**A**) open inactive and (**B**) closed active conformation. Upon substrate binding, the NS2B region (colored purple) spanning residues 63 to 88 undergoes conformational changes around the hinge at residue 63, subsequently interacting with the region adjacent to Ala125, which is located between the 120 s and 150 s loops. Ala125 is depicted as spheres, while the catalytic triad is illustrated as sticks. NS3 is in yellow in (**A**) and green in (**B**). The crystal structure of DENV3 NS2B-NS3 protease (**B**) is complexed with the substrate-like, active-site inhibitor **Bz-nKRR-H** (represented as white sticks). Figure taken from Yildiz M. et al. ACS Chem Biol. 2013, 8(12), 2744–2752 [75].

Furthermore, NS2B-NS3 exists in two distinct conformations (Figure 5), an open, inactive conformation and a closed, active conformation. Competitive orthosteric inhibitors are studied in the closed, active conformation of NS2B-NS3 (Figure 5B), which is formed upon binding with a substrate-like molecule in the active site. The impact of orthosteric non-competitive inhibitors on conformational changes remains unclear. An allosteric site has been identified in the NS3 subunit behind the active site (Figure 5), around Ala125. Allosteric inhibition hinders the NS2B cofactor to close around the NS3 subunit, impeding the formation of the active site. It is possible that allosteric inhibition may be achieved also by binding to different regions of the open conformation. While targeting the orthosteric site presents challenges due to the shallow surface of the S1–S4 subsites, structural rationalization of allosteric inhibition is also difficult because of the dynamic nature of the drastic conformational change between the inactive and active forms [75,76,77,78].

This review aims to provide a comprehensive overview of small-molecule drug development targeting DENV, ZIKV, and WNV, as these represent the orthoflaviviruses that cause the highest health burden to humans. We focus on preclinical and clinical results for compounds inhibiting viral replication to determine the impact of NS2B-NS3 inhibition on anti-orthoflaviviral drug development. We systematically examine clinical trials registered on ClinicalTrials.gov that assess the safety and efficacy of various viral replication inhibitors in human patients and report their preclinical efficacy and mode of action. Additionally, we discuss allosteric and orthosteric drugs inhibiting the NS2B-NS3 protease with proven in vivo efficacy. Because of the limited number of competitive orthosteric viral protease inhibitors reported with in vivo efficacy, we also explore in vitro-tested competitive NS2B-NS3 protease inhibitors exhibiting cellular antiviral activity. This review offers a detailed analysis of the anti-orthoflaviviral drug development pipeline as of June 2024, emphasizing viral NS2B-NS3 protease inhibitors. It should be noted that such inhibitors, including the ones for which no in vivo efficacy has yet been reported and which are not discussed here, have recently been reviewed [79]. 

## 6. Small-Molecule Therapeutics in Clinical Trials

**Figure 6:**
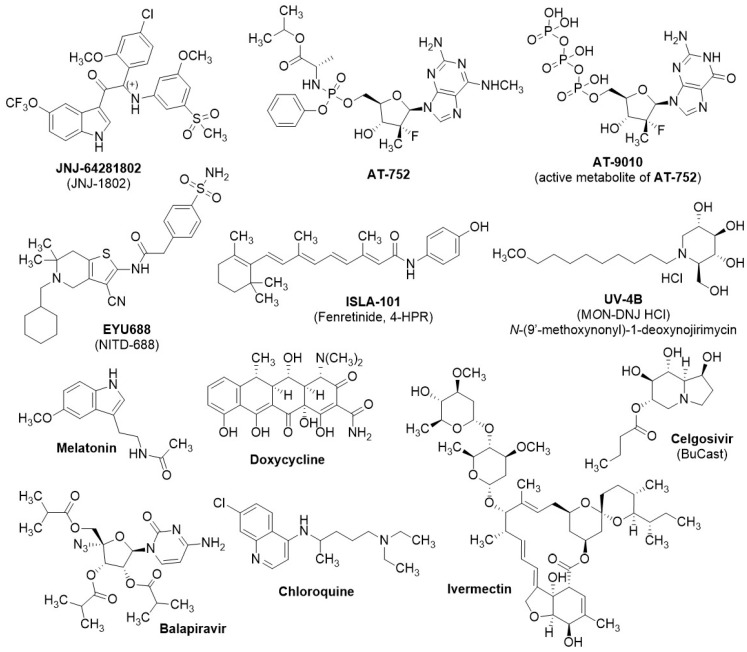
Chemical structures of viral replication inhibitors of clinical relevance from the literature. References and biological activities are in Table 1.

A comprehensive search was conducted on ClinicalTrials.gov, the EU Clinical Trials Register, and the international traditional medicine clinical trial registry (ISRCTN) using the terms “Drug NON Biologic” or “NOT vaccine” in conjunction with the target virus (DENV, ZIKV, or WNV) to identify pertinent therapeutic candidates. All retrieved results were evaluated for relevance and subsequently incorporated into the study. A total of eleven compounds were identified that inhibit orthoflaviviral growth and are either registered on ClinicalTrials.gov or announced for clinical trials by their respective sponsors. Figure 6 presents an overview of these agents, and in Table 1 their most significant clinical and preclinical data are reported. Based on the findings from our study, only one small-molecule therapeutic against DENV or orthoflaviviral infections has demonstrated antiviral efficacy in clinical development thus far, although several compounds exhibited antiviral efficacy in preclinical animal models. To assess the in vivo efficacy of potential anti-orthoflavivirals, a variety of infected murine models were employed, including A129 and AG129 mouse strains, Balb/c, ICR suckling, Chinese Kunming, and Atg16/1 HM mice. The AG129 mouse model, which lacks both type I (IFN-α/β) and type II (IFN-γ) interferon receptors, is especially valuable for antiviral drug development against DENV and ZIKV. This is due to its heightened susceptibility to viral infections, making it the most widely used model in this area of research [80,81]. In contrast, the A129 mouse model is deficient solely in the type I interferon (IFN-α/β) receptor and has mostly been used in ZIKV infection models [82]. The in vivo experiments focused on evaluating generally two primary outcomes: survival rates (SR) and viral load reduction in drug-treated groups compared with vehicle control groups. The highest DENV viremia level is measured around days 3 and 4 post-infection in the AG129 model, and it is reported here as peak viremia. The viral load during peak viremia is generally quantified through viral RNA copies or viral pFU per organ. In common animal trials, the treatment is started either the same day as or prior to infection (prophylactic regimen, PR in Table 1), or 24–72 h post infection (therapeutic regimen, TH in Table 1). In most cases, differences in viremia were not observed in treatments started >48 h post infection.

It is important to note that the pathogenesis of orthoflaviviral infection in those animal models is different from the timeline observed in humans infected through a mosquito bite. For instance, common symptoms of dengue are shown after 4–7 days of incubation, and DENV can be detected only slightly before. The symptomatic (febrile) stage lasts for about 5–7 days, and while it is self-resolving in most cases, it can escalate in some toward a critical phase, resulting in a hemorrhagic fever or shock syndrome that can be life-threatening [83]. An anti-DENV therapeutic treatment (wt-TH in Table 1) should be administered in the early febrile window to show detectable efficacy and potentially prevent internal bleeding and plasma leakage. Most recent studies require the patient to show symptoms for <48 h to be included in a therapeutic regimen. Sampling and fast inclusion in clinical trials make the recruitment of eligible patients with confirmed DENV often difficult. A prophylactic treatment regimen would instead be administered prior to a possible infection and could potentially serve as a preventive treatment in people at risk (wt-PR in Table 1). In some recently developed dengue human infection models (DHIM in Table 1, or dengue human challenge) [84,85], the participants are inoculated with an attenuated virus after the start of a prophylactic regimen of the drug under investigation.

**Table 1:** Orthoflaviviral replication inhibitors of clinical relevance.

			Preclinical Data		Clinical Data			
Name	Target	Cellular EC_50_ Virus (Cell Line)	Peak Viremia Reduction or %SR, Drug Regimen ^[a]^, Dosage, Route ^[b]^ (Animal Infection model) ^[c]^	Trial Identifier	Phase (Study Type) ^[d]^	Year ^[e]^	Status/Outcome	Ref.
**JNJ-64281802**	NS3–NS4B	0.06–1.4 nM DENV1-4 (Multiple)	≤LLOD, PR, 3 mg/kg, SC (DENV1/2, NHP) ≤LLOD, PR, 2 mg/kg, IP (DENV2 RL, AG129) <1-log, TH (4d p.i.) ^[f]^ 60 mg/kg, IP (DENV2 RL, AG129)	NCT05201937 NCT04906980 NCT05048875 NCT05201794 NCT04480736	I II (wt-TH) II (DHIM-DENV3) II (wt-PR) II (DHIM-DENV1)	2023 2024 2024 2025 2027	Safe Terminated ^[g]^ Recruiting Recruiting Suspended	[86,87]
**AT-752**	Viral RNA polymerase	0.49/0.77 μM ^[h]^ DENV2/3 (Huh-7)	≤1-log, PR, 1000–500 mg/kg, PO (DENV D2Y98P, AG129)	NCT04722627 NCT05366439 NCT05466240	I I (DHIM) II (wt-TH)	2021 2023 2023	Safe Terminated ^[i]^ Terminated ^[i]^	[88,89]
**EYU688**	NS4B	8–38 nM DENV1-4 (Vero)	4.3-log, PR, 100 mg/kg, PO (DENV2 RL, AG129) ≤-log, TH (2d p.i.), 30 mg/kg, PO (DENV2 RL, AG129)	NCT06006559	II (wt-TH)	2026	Recruiting	[90]
**ISLA-101**	Host nuclear import inhibitor, NS5 entry	1.3–2.4 µM DENV1-4 (Huh-7)	70% SR, PR, 20 mg/kg, PO (DENV2 S221, AG129) ^[j]^	n.a.	I II (TH and PR)	2022 2023 ^[k]^	Safe ^[k]^ Announced	[91,92]
**UV-4B**	Host ER glucosidases	2.1–87 µM DENV1-4 (Vero)	100% SR, PR, 40 mg/kg, PO 90% SR, TH (2d p.i.), 40 mg/kg, PO (DENV2 S221AG129) ^[l]^	NCT02061358 NCT02696291	I I	2015 2017	Safe (1000 mg) Terminated ^[m]^	[93,94]
**Melatonin**	Host anti-inflammatory factors/viral proteins (NS3)	140–200 µM DENV2 (Huh-7 EA.hy.926, A549, U937)	SR 69%, PR, 500 µg/kg (WNV WN-25, CD1)	NCT05034809	II (wt-TH)	2022	Not yet recruiting	[95,96]
**Doxycycline**	NS2B-NS3	40 µM DENV2 (Vero)	n.a.	n.a. n.a. CTRI/2021/09/036661 CTRI/2018/01/011548	II (wt-TH) II (wt-TH) II (wt-TH) II (wt-TH)	2015 2022 2023 2019	Reduction in inflammatory markers n.a.	[97,98,99]
**Celgosivir**	Host ER glucosidases	0.22–0.65 µM DENV1-4 (BHK-21)	16.5-fold, PR, 50 mg/kg, PO <1 log, TH (2d p.i.), 50 mg/kg, PO (DENV EDEN2, AG129)	NCT01619969 NCT02569827	I/II (wt-TH) I/II (wt-TH)	2013 2019	No efficacy Withdrawn	[100,101,102]
**Ivermectin**	Host nuclear import inhibitor, NS5 entry	1.2–1.6 µM DENV1-4 (BHK-21)	n.a.	NCT03432442 NCT02045069	II (wt-TH) ^[n]^ II/III (wt-TH) ^[n]^	2020 2016	No efficacy Withdrawn	[103,104]
**Balapiravir**	Viral RNA polymerase	1.9–11 µM DENV1-4 (Huh-7)	n.a.	NCT01096576	I (wt-TH)	2011	No efficacy	[105]
**Chloroquine**	Virus assembly	1.7–2.7 µM ZIKV MR766 (Vero) ^[o]^	<LLOD ^[p]^, PR, 25 mg/kg, PO 75% ^[p]^, TH (1d p.i.), PR, 25 mg/kg, PO 25% ^[p]^, TH (2d p.i.), PR, 25 mg/kg, PO (DENV2 NGC, NHP)	NCT00849602 ISRCTN38002730	I/II (wt-TH) ^[n]^ I/II (wt-TH) ^[n]^	2009 2010	Unknown No efficacy	[106,107,108,109]

Abbreviations: n.a. = not available; LLOD, lower limit of detection; SR: survival rate; ^[a]^ PR: prophylactic; TH: therapeutic (d p.i.: days post-infection). ^[b]^ Administration routes: PO, oral administration; SC, intradermal administration, IP: intraperitoneal administration. ^[c]^ AG129 is a murine model; NHP, non-human primate; CD1, a murine model. ^[d]^ wt-TH: patients with dengue fever (<48 h fever onset) are administered the drug under investigation with a therapeutic treatment; DHIM virus: dengue human infection model, attenuated infection of healthy participants after a prophylactic treatment; wt-PR: prophylactic study, healthy participants are administered the drug, and the protection against wild-type dengue is assessed. ^[e]^ Latest relevant trial update/est. completion year. ^[f]^ Attenuated viral regimen, peaks through days 4 to 6. ^[g]^ Due to the small number of enrolled participants, data collection and analysis was not performed for the efficacy; thus, only safety analysis data were reported. ^[h]^ Data relative to the active metabolite AT-9010. ^[i]^ Sponsor decision to deprioritize the dengue program. ^[j]^ Efficacy proven also against ZIKV: 1-log peak viremia reduction, following 60 mg/kg IP, (ZIKV MR-766, AG129) [110,111]. ^[k]^ Details and results of the trial announced but not disclosed in peer-reviewed journals. ^[l]^ In vitro DENV-2 S221-infected BHK cells EC_50_ = 39 µM. ^[m]^ Product development halted for business reasons. ^[n]^ Administration of treatment < 72 h before symptoms onset. ^[o]^ Cellular efficacy against DENV has been proven [106]; however, in the literature only the EC_50_ in ZIKV-infected cells was reported [109]. This EC_50_ value is reasonably in the same range of the reported cellular efficacy observed against DENV. ^[p]^ Qualitative assessment, % of animals not presenting infection markers.

In this review, only small molecules that showed direct viral growth inhibition were included. Of the eleven small molecules that are in or have completed clinical trials and that we identified as relevant to our study (Figure 6), five (**JNJ-64281802**, **AT-752**, **EYU688**, **ISLA-101**, and **UV-4B**) have recently entered or completed phase I clinical trials, and their potential clinical antiviral efficacy remains to be determined. **JNJ-64281802** (**JNJ-1802**, renamed mosnodenvir) has recently shown promising clinical efficacy in a prophylactic treatment against attenuated DENV3 infection (DHIM) in a phase II clinical trial [112] (announcement not peer-reviewed). Four (**Ivermectin**, **Celgosivir**, **Balapiravir**, and **chloroquine**) completed their clinical trials between 2010 and 2020, but none exhibited clinical efficacy against DENV. For the phase II clinical trial of **melatonin**, as a coadjutant to standard care in dengue fever, recruitment has not yet begun. **Doxycycline** is the only NS2B-NS3 inhibitor currently undergoing clinical investigation; however, its mechanism of action and clinical efficacy as a viral growth inhibitor have not yet been established.

**JNJ-64281802** [113] is at the cutting edge of antiviral drug development against DENV, showing favorable oral bioavailability, pharmacokinetics, and safety profile in a phase I clinical trial in healthy patients (NCT05201937) [86] and is being registered in four additional phase II efficacy trials (NCT04906980, NCT05048875, NCT05201794, and NCT04480736). Among the phase II clinical trials, two have been halted due to patient recruitment issues deriving from the 2020–2022 COVID pandemic. It is an NS3-NS4B inhibitor, which hinders formation of the viral replication complex (Figure 1, step 6). **JNJ-64281802** shows subnanomolar antiviral efficacy against DENV (EC_50_ = 0.06–1.4 nM, depending on virus strain and cell line) and potent dose-dependent efficacy in vivo (viral RNA levels ≤ LLOD (lower limit of detection) in infected AG129 mice and NHPs (non-human primates), following resp. 6 mg/kg IP (intraperitoneal) and 3 mg/kg SC (subcutaneous)) [87]. Notably, the efficacy of the compound wanes in preclinical murine models after delayed administration post-infection. Preliminary data released by Johnson & Johnson Innovative Medicine (formerly known as Janssen Pharmaceuticals) of the phase IIa clinical trial NCT05048875 showed promising human efficacy data in a prophylactic DENV3 human challenge trial (DHIM): participants received a daily dose of either **JNJ-64281802** or a placebo over a span of 26 days. On day five, they were inoculated with attenuated DENV3. Sixty percent of the high-dose antiviral recipients showed no viral RNA in their blood throughout the study. Conversely, detectable viral RNA appeared in all placebo-treated subjects within five days post-exposure. Those administered low to medium doses of the antiviral showed evidence of viral RNA but with a delayed onset relative to the placebo group [112]. In vitro viral kinetic modeling of **JNJ-64281802** favorably describes NS3-NS4B inhibition as a therapeutic target against DENV infection in a prophylactic regimen, blocking the transition of infected cells to infectious ones [114]. 

**AT-752** is a protected guanosine nucleotide analog (pro-drug); its metabolite **AT-9010** inhibits RNA synthesis by acting as an RNA chain terminator, targeting two NS5-associated enzyme activities, the RNA 2′-O-MTase and the viral RNA polymerase [115]. **AT-9010** showed submicromolar efficacy in infected cells (DENV2- or DENV3-infected Huh-7 cells, resp. EC_50_ = 0.49 μM and 0.77 μM) [89], while **AT-752** showed limited efficacy in DENV2-infected AG129 mice (≤1-log peak viremia reduction, following 1000–500 mg/kg PO (oral administration)) [89], especially if compared with **JNJ-64281802**. **AT-752** favorably passed a phase I clinical trial (NCT04722627), where it was shown to be safe in the therapeutic regimen intended to treat DENV infection [88]. Two phase II clinical trials were initiated, but both were prematurely terminated and the development halted.

**EYU688** is an NS4B inhibitor developed by Novartis, and it is the second, after **JNJ-64281802**, most effective compound (DENV1-4 EC_50_ = 8 to 38 nM in Vero cells, up to 4.2-log peak viremia reduction in AG129 mice, following 100 mg/kg PO), among those in Table 1 still actively in development [90]. **EYU688** is now initiating a phase II clinical trial (NCT06006559) in dengue patients, which is scheduled to be completed in 2026.

**ISLA-101** is an NS5 nuclear transport inhibitor in development by Island Pharmaceuticals, and, compared with **EYU688**, showed significantly lower preclinical efficacy against DENV [91,92]. Island Pharmaceuticals announced that **ISLA-101** entered phase II trials in DENV-infected patients in 2023; however, the absence of trial registration makes it challenging to understand its full potential. Recently, Island Pharmaceuticals announced that the compound will be trialed also in a prophylactic regimen [116]. **UV-4B** is a host glucosidase inhibitor, which showed good preclinical efficacy (90–100% SR in AG129 mice following 20 mg/kg PO) and favorably passed a phase I clinical trial (NCT02061358), deeming it as safe [93,94]. Notably, **UV-4B** shows significant efficacy also after delayed administration in the survival model. **UV-4B** was scheduled to initiate an additional phase I trial (NCT02696291); however, its development was halted and the trial terminated.

**Melatonin** is a hormone primarily produced by the pineal gland in the brain. Its production increases in response to darkness and decreases with light exposure, playing a crucial role in regulating circadian rhythms [117]. **Melatonin** is currently undergoing one phase II clinical trial (NCT05034809) as a coadjutant to standard care in dengue fever with warning signs. Although the trial was expected to be completed in 2022, patient recruitment has not yet begun. **Melatonin** exhibits antiviral properties against several orthoflaviviruses in vitro, such as DENV, ZIKV, and JEV, and WNV also in vivo. In vitro antiviral activity is often reported in the high-micromolar range (> 100 µM). **Melatonin** exerts its antiviral activity by inhibiting various host anti-inflammatory mechanisms and possibly viral proteins. It inhibits DENV production via the activation of the sirtuin 1-mediated interferon pathway, modulating the transcription of antiviral genes and suppressing viral replication, showing EC_50_ ranging from 140 to 200 µM in Huh-7, EA.hy.926, A549, and U937 cells [95]. Notably, a parallel screening study showed limited (EC_50_ > 500 µM) antiviral activity of **melatonin** in HEK293T/17 and HepG2 cells [118]. **Melatonin** inhibits ZIKV replication in Vero and SK-N-SH cells, possibly by interfering with NS3 or NS3 production [119]. For JEV, melatonin inhibits viral replication and reduces neurotoxicity by modulating the calcineurin-autophagy pathway [120]. In WNV infections, **melatonin** reduces viremia and delays the onset of disease in stressed mice by modulating immune responses and reducing inflammation [96].

**Doxycycline** is a tetracycline antibiotic with broad-spectrum antimicrobial properties and anti-inflammatory activity. It has demonstrated significant antiviral activity against dengue virus (DENV) in vitro, with an EC_50_ = 40 µM in Vero cells infected with DENV2. It inhibits DENV2 NS2B-NS3 with an IC_50_ value of 52.3 ± 6.2 µM at 37 °C (normal human temperature) and 26.7 ± 5.3 µM at 40 °C (high fever temperature) [121]. **Doxycycline** seems to exert its antiviral activity during the early to mid-stages of infection, interfering with viral entry and replication processes. Despite showing NS2B-NS3 inhibition, further studies are needed to understand its exact mode of action. It has undergone three phase II clinical trials, but no viral load reduction was recorded in any of them. It appears to improve the prognosis of severe DENV patients and to reduce the inflammation markers associated with the disease; further investigation of its mode of action and clinical efficacy is required [97,98,99]. Currently, it is registered in an additional phase II trial in India (CTRI/2018/01/011548), which should have been concluded in 2019, but to date no public results are available. **Doxycycline** inhibited NS2B-NS3 of ZIKV even more strongly than that of DENV, with IC_50_ values of 9.9 and 5.3 µM at 37 and 40 °C, respectively; at 20 µM it gave approximately 50% reduction in ZIKV replication in human skin fibroblasts, and at 40 µM it almost eliminated the cytopathic effect [122].

Among the compounds that did not show clinical efficacy, **celgosivir** is worth noting, because it drew particular attention due to its promising early preclinical efficacy [80]. It exerts its antiviral activity through host ER (endoplasmic reticulum) α-glucosidase inhibition, similarly to **UV-4B**, resulting in misfolding of several viral proteins such as E, prM, and NS1, which are essential for viral RNA replication [100]. **Celgosivir** showed submicromolar pan-serotype (DENV1-4) antiviral efficacy in infected cells (EC_50_ = 0.22–0.68 µM) [100] and high antiviral efficacy in DENV2-infected AG129 mice (from 2.4- to 16.5-fold peak viremia reduction, following 50 mg/kg PO) [101]. However, it did not show clinical efficacy in humans against DENV in a phase I/II trial in 2013 (NCT01619969) [102], and a subsequent phase I/II trial, scheduled to conclude in 2019 (NCT02569827), where it should have been compared with treatment with modipafant, was withdrawn. No in vivo preclinical data are available for **ivermectin** and **balapiravir**, but, being repurposed drugs, they are safe for humans. They showed promising antiviral efficacy in cells (resp. EC_50_ = 1.2–1.6 µM [103] and 1.9–11 µM [114]) but no clinical efficacy (resp. NCT03432442 and NCT01096576). **Ivermectin** is an antiparasitic drug used to treat intestinal roundworms, lice, and scabies, and its antiviral efficacy against DENV was ascribed to inhibition of the nuclear transport of NS5 [103,123], similarly to **ISLA-101**. Although **ivermectin** did not show clinical efficacy in the treatment of DENV in humans (NCT03432442), its antiviral pharmacology is still being researched [124], and it seems to show promise in combination with the cholesterol-lowering drug atorvastatin [125,126]. **Balapiravir** is a nucleoside analog that was initially developed against HCV, and it supposedly exerts antiviral activity in vitro through inhibition of the viral RNA polymerase [104], as established for **AT-9010**/**AT-752**. Since the mechanism through which **balapiravir** exerts anti-orthoflaviviral activity (as observed in cells) is solely inferred from its known action against HCV, and there is no direct evidence confirming that it would function through the same mechanism in DENV, it is crucial not to dismiss the potential for RNA polymerase inhibition to yield clinical efficacy. **Chloroquine** is an approved antimalarial drug that showed pan-serotype micromolar cellular efficacy, and it is thought to interfere with host machinery, resulting in the inhibition of viral assembly [105]. Despite showing very potent antiviral efficacy in NHPs at very low dosing (peak viremia ≤ LLOD, 5 mg/kg, PO), it failed to show clinical antiviral efficacy in a phase II clinical trial [106]. It is plausible that the specific antiviral mechanisms of **melatonin**, **ivermectin**, **celgosivir,** and **chloroquine** that account for their observed antiviral efficacy in animal models do not translate into clinical antiviral efficacy in human patients, possibly because they are characterized by inhibition of and/or interaction with host pathways, which may vary between humans and the animal models. On the contrary, **JNJ-64281802**, **AT-752,** and **EYU688** exert their antiviral activity through inhibition of the viral machinery (resp. NS3-NS4B, viral RNA polymerase, and NS4B), directly hindering viral RNA replication. While host pathways may vary among different species, inhibition of viral machinery may hopefully allow the translation of preclinical antiviral efficacy in animals to clinical antiviral efficacy in humans. Conversely, the in vivo anti-orthoflaviviral preclinical efficacy of **ISLA-101**, which inhibits NS5 host nuclear transfer in a manner similar to ivermectin, and of UV-4B, which exerts activity through host ER glucosidases’ inhibition, akin to **celgosivir**, might not translate to clinical efficacy in humans. Several compounds scrutinized herein (**AT-752**, **UV-4B**, **ivermectin**, **celgosivir**, **chloroquine,** and **balapiravir)** either did not complete phase II efficacy trials (studies terminated or withdrawn) or did not show clinical efficacy in dengue patients after a therapeutic regimen, even though they performed favorably in preclinical orthoflaviviral infection animal models. It is important to note that most of those compounds showed already waning efficacy after delayed administration post-infection in the same animal models. It cannot be ruled out that the animal models employed in anti-orthoflaviviral drug development (e.g., DENV-infected AG129 mice or NHPs) may not accurately represent DENV infection in humans, especially in a therapeutic application. The preliminary data from the phase IIa clinical trial of **JNJ-64281802** suggest that current animal models may approximate human DENV infection at least in a prophylactic regimen of the drug under investigation. Nevertheless, a conclusive assessment hinges on the release of more comprehensive data, particularly because viral RNA was still detectable in 40% of participants receiving the highest dose, contrary to what was observed in the animal models. These results highlight the relevance of developing novel anti-orthoflavivirals based on the inhibition of diverse viral targets, such as NS2B-NS3 inhibition, which is so far underrepresented in the clinical settings.

## 7. Small-Molecule NS2B-NS3 Protease Inhibitors with In Vivo Efficacy

**Figure 7:**
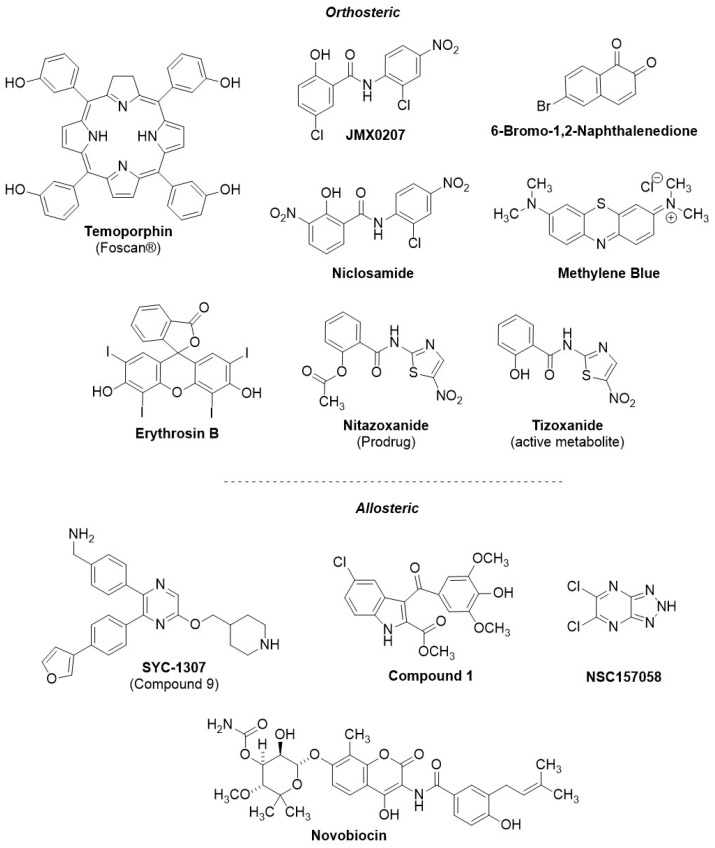
Chemical structures of NS2B-NS3 inhibitors with reported in vivo anti-orthoflaviviral efficacy from the literature. References and biological data for orthosteric inhibitors in Table 2 for allosteric inhibitors in Table 3.

In order to understand the preclinical developmental status of NS2B-NS3 viral protease inhibitors, we performed a systematic search using PubMed, Reaxys, Google Scholar, and ChemBL. Compounds were selected cross-referencing orthoflaviviral NS2B-NS3 protease inhibition with demonstrated in vivo anti-orthoflaviviral efficacy in an animal model, identifying 12 relevant compounds meeting these criteria (Figure 7). Notably, the in vivo antiviral efficacy of the vast majority of those viral protease inhibitors is reported against ZIKV infection models, although they also show in vitro potency against other orthoflaviviruses. Only for **niclosamide** was the antiviral efficacy against DENV in vivo reported. 

The identified NS2B-NS3 inhibitors were further classified based on their reported mode of inhibition, either orthosteric or allosteric (resp. Table 2 and Table 3, structures of both categories in Figure 7). In the animal studies reported for both sets of compounds, the drug under investigation was administered at the same time as the viral infection, so we define the drug regimen as prophylactic in all cases.

The orthosteric inhibitors identified in Table 2 were principally discovered in libraries of repurposed approved drugs, which were screened for in vitro NS2B-NS3 protease inhibition. Interestingly, among the orthosteric NS2B-NS3 inhibitors with demonstrated in vivo efficacy, only one compound, **6-bromo-1,2-naphthalenedione**, was reported to be a competitive inhibitor. All other compounds listed in Table 2 have demonstrated binding to the active site of the viral protease; however, they are reported to exhibit non-competitive inhibition behavior. **6-Bromo-1,2-naphthalenedione** is a selective ZIKV NS2B-NS3 protease inhibitor (IC_50_ = 67 µM), identified after in silico structure-based screening, and is approved for the treatment of influenza. The binding of the compound to the protease has been rationalized through in silico docking. Its specificity for ZIKV protease, compared with other orthoflaviviruses, appears to result from its interaction with specific residues in the S3–S4 subsites of the ZIKV protease. **6-Bromo-1,2-naphthalenedione** showed antiviral efficacy against ZIKV in infected cells (EC_50_ = 1 µM) and in ZIKV-infected AG129 (20–60% SR, up to 3.7-fold peak viremia reduction following 50 µg IV) [135]. Various aspects of **6-bromo-1,2-naphthalenedione** are still unclear; for instance, there is a significant discrepancy between its low potency against the protease and its high efficacy against the virus as well as its reported competitive mechanism combined with high selectivity against ZIKV. The remaining identified orthosteric inhibitors showing in vivo efficacy (viz. **temoporfin**, **JMX0207**, **niclosamide**, **methylene blue,** and **erythrosin B**) inhibit the NS2B-NS3 protease via a non-competitive mechanism. Preclinical data suggest that **temoporfin** is the most promising orthosteric NS2B-NS3 protease inhibitor (NS2B-NS3 IC_50_ = 1.1 µM, cellular infection EC_50_ = 0.010–0.024 µM), exhibiting a strong reduction in peak viremia at low doses (2-log peak viremia reduction in ZIKV-infected Balb/c mice following 1.6 mg/kg IP). **Temoporfin** is an already-approved pharmaceutical in oncology treatment, and, while it is safe in humans, it is not orally bioavailable [127]. **JMX0207**, **niclosamide**, **methylene blue**, **erythrosin B**, and **nitazoxanide** display relatively limited in vivo efficacy compared with **temoporfin** and **6-bromo-1,2-naphthalenedione**, exhibiting < 2-log peak viremia reduction in vivo. Among those, **methylene blue** demonstrates the most significant reduction in peak viremia in vivo (1.7-log reduction in peak viremia following 100 mg/kg PO), while inhibiting in vitro the DENV protease in the micromolar range (IC_50_ = 9 µM) and DENV infection in cells in the submicromolar range (EC_50_ = 0.36 µM). Although **niclosamide** had been previously identified as an NS2B-NS3 inhibitor [127], later studies involving a replicon assay revealed that it may instead exert its antiviral activity through endosomal acidification, retarding the DENV infection rather than inhibiting NS2B-NS3 activity [131]. Considering the preclinical findings, **6-bromo-1,2-naphthalenedione**, **temoporfin,** and **methylene blue** appear to show the most significant antiviral efficacy in vivo among orthosteric NS2B-NS3 inhibitors. Notably, the **temoporfin** study was published in 2017 [127], and no following studies have been disclosed until now, suggesting that it is unlikely to show promising efficacy. **6-Bromo-1,2-naphthalenedione**, **temoporfin**, and **methylene blue** show significantly lower in vivo antiviral efficacy compared with **JNJ-64281802** and **EYU688**, and hence, their clinical relevance may be limited; however, they may represent viable scaffolds for further development of analogs with improved efficacy.

Additionally, we identified four relevant allosteric inhibitors of the NS2B-NS3 protease with proven in vivo efficacy (Table 3). Three (**SYC-1307**, **compound 1,** and **NSC157058**) are novel compounds, and one (**novobiocin**) is a repurposed approved drug. **SYC-1307**, the most promising among these, belongs to a novel series of 2,5,6-trisubstituted pyrazine derivatives, and it is a submicromolar allosteric inhibitor of the orthoflaviviral NS2B-NS3 protease (determined in DENV, ZIKV, WNV, IC_50_ = 0.2–0.8 μM), with submicromolar anti-orthoflaviviral efficacy in cells (EC_68_ = 0.3–0.6 μM), correlating with significant reduction in ZIKV peak viremia in vivo (96–98% reduced viral RNA following 15–30 mg/kg IP) [140,141]. **NSC157058** also showed similar efficacy in vivo against ZIKV (10-fold peak viremia reduction, 100% SR, following 30 mg/kg PO) and submicromolar orthoflaviviral NS2B-NS3 inhibition (IC_50_ = 0.8 µM); however, it demonstrated limited cellular antiviral efficacy (EC_50_ = 50 µM) [78]. **Compound 1** and **novobiocin** also exhibited in vivo efficacy against ZIKV (100% SR, resp. 1 mg/kg IP and 100 mg/kg SC); however, they showed limited in vitro inhibitory potency against NS2B-NS3 (resp. IC_50_ = 158 and 14 µM) and limited antiviral efficacy in infected cells (EC_50_ = 14 and 43 µM) [142,143,144] compared with **SYC-1307**. Difficulties in correlating the in vitro potency and efficacy with the in vivo efficacy may hamper the structure-optimization process. **SYC-1307**, bearing strong correlation between potency and efficacy (both in vitro and in vivo), an extensive SAR, broad-spectrum anti-orthoflaviviral potency in vitro, and demonstrated potent antiviral efficacy against ZIKV in vivo, represents the most promising compound among the NS2B-NS3 protease inhibitors (orthosteric and allosteric) with reported in vivo efficacy in the literature. Furthermore, from our study a substantial disparity between non-competitive and competitive inhibitors (1 competitive against 11 non-competitive) emerges, underscoring the challenges in developing competitive NS2B-NS3 inhibitors.

## 8. Small-Molecule NS2B-NS3 Orthosteric Competitive Inhibitors with Cellular Efficacy

**Figure 8:**
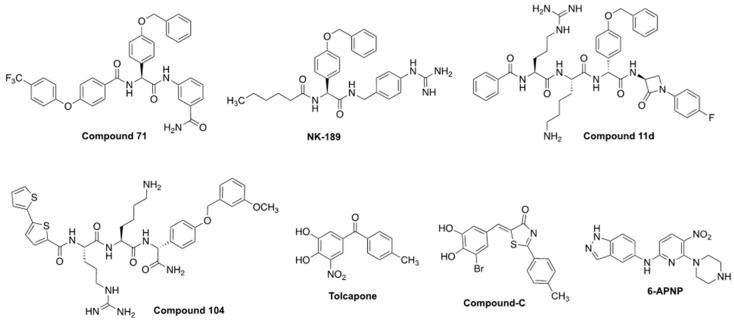
Competitive NS2B-NS3 inhibitors with potent cellular efficacy (EC_50_ < 20 µM). References and biological data in Table 4.

In the following section, we summarize the recent advancements in designing competitive NS2B-NS3 protease inhibitors that have undergone in vitro testing against DENV. Until now, no competitive viral protease inhibitors with in vivo efficacy against orthoflaviviral infections have been reported, with the exception the aforementioned case of **6-bromo-1,2-naphthalenedione** against ZIKV. To encompass all pertinent drug development, we will examine seven competitive NS2B-NS3 protease inhibitors with EC_50_ values below 20 μM (structures in Figure 8 and biological data in Table 4). These compounds were primarily identified through a comprehensive search in the EMBL-EBI ChEMBL database, which included all compounds demonstrating competitive NS2B-NS3 inhibition and exhibiting cellular efficacy below the 20 µM threshold. Additional competitive inhibitors meeting the established criteria were identified; however, they are less-potent analogs of the compounds discussed in Figure 8. For instance, **mb-53,** shown in Figure 4 in the introduction, is an analog developed parallelly to **compound 104** and having slightly lower viral protease inhibitory potency and cellular efficacy. Moreover, numerous competitive NS2B-NS3 peptide-like inhibitors are known but not discussed in this section due to their low (EC_50_ > 20 μM), absent, or unreported cellular efficacy data. As previously mentioned in the introduction, challenges in structure-based drug design approaches for NS2B-NS3 inhibition can be attributed to the majority of the peptide-like competitive NS2B-NS3 inhibitors reported in the literature. These inhibitors often possess positive charges that negatively affect cellular efficacy [59,60,72]. Furthermore, biochemical assay conditions do not precisely replicate the viral proteolytic processes that occur within cells, often resulting in a weak correlation between biochemical potency and cellular efficacy. 

Nevertheless, relentless medicinal and synthetic chemistry efforts, principally driven by reducing the cationic and peptidic character of substrate-like inhibitors such as **Bz-nKRR-NH_2_** and optimizing assay conditions to better replicate NS2B-NS3 inhibition in vitro, allowed the development of compounds such as **NK-189** [59] followed by its optimized scaffold **compound 71** [61], both showing submicromolar cellular efficacy (resp. EC_50_ = 0.89 and 0.24 µM). Between **Bz-nKRR-NH_2_** and **NK-189,** several iterations of peptide-like derivatives, such as **cn-716**, **mb-53** (both shown in Figure 4), and **compound 11d** were explored, also to structurally characterize the viral protease. Notably, **compound 11d** is the only reported competitive covalent NS2B-NS3 protease inhibitor showing relevant cellular efficacy [145]. However, proteolytic studies revealed that the fragment bearing the covalent warhead is excised by the protease, resulting in an analog of **mb-53**, which is thought to be actually responsible for its antiviral efficacy [145].

The development of **NK-189** and **compound 71** was facilitated by the establishment of a replicon assay that enabled the selection of viable antivirals in a virus-free cellular environment while specifically targeting the NS2B-NS3 inhibition pathway [59,61]. **Compound-C** and **tolcapone** were identified in a high-throughput screening of 120,000 commercially available compounds as potential inhibitors of DENV, which were evaluated using replicon, plaque, and cytotoxicity assays [146]. They are repurposed, commercially available drugs, which lack peptide features, and, together with **6-bromo-1,2-naphthalenedione,** they are the only reported small-molecule NS2B-NS3 competitive inhibitors with relevant cellular efficacy (resp. EC_50_ = 8.97 and 2.03 µM). The activities of **Compound-C** and **tolcapone** correlate with potent pan-serotype viral protease inhibition (resp. IC_50_ = 2.94–4.06 and 0.64–1.15 µM) [146]. The research identifying **Compound-C** and **tolcapone** was conducted in 2016; considering the time that has passed, it is unlikely that these molecules exhibited adequate efficacy in animal models. **6-APNP** was identified as an orthosteric inhibitor, notably, after a structure-based HTS screening in silico aimed to identify NS2B-NS3 inhibitors [147]. Its binding to the protease could be well-rationalized in silico, and the compound showed micromolar to submicromolar antiviral efficacy against DENV4 and ZIKV (respectively, EC_50_ = 7.1 and 0.69–5.0 µM), and similar viral protease inhibitory potency (IC_50_ = 1.5 µM) [147]. **Tolcapone**, **compound-C,** and **6-APNP** are less potent than peptide derivatives such as **compound 71**; however, they present more drug-like features, which can ease their development and lead to an improved pharmacokinetic profile in vivo. Although direct analogs with cellular efficacy have not yet been documented, **tolcapone**, **compound-C,** and **6-APNP** may serve as scaffolds in the development of future non-peptide NS2B-NS3 inhibitors.

## 9. Conclusions

In this study, we systematically analyzed the scientific literature and publicly available clinical data to assess the current state of drug development against orthoflaviviral infections, with a particular focus on viral replication inhibitors. Moreover, we sought to understand the role of NS2B-NS3 inhibition in this context. Our analysis began with clinical trials involving small molecules, followed by a review of NS2B-NS3 protease inhibitors exhibiting in vivo antiviral efficacy, and concluded with a focus on competitive NS2B-NS3 inhibitors. It is concerning that numerous compounds with demonstrated in vivo efficacy failed to exhibit clinical antiviral efficacy, raising doubts regarding the accuracy of the animal models employed in anti-orthoflaviviral drug development in representing orthoflaviviral infections in humans. This may be explained by differences in the pathogenesis of dengue between humans and animal models and by possible variations in immunological response and drug metabolism (pharmacokinetics) between species. More importantly, the assessment of the efficacy of a therapeutic treatment against wild-type dengue in a clinical setting (wt-TH in Table 1, treatment administered < 48 h of the symptoms’ onset) may be hampered by the fact that most compounds already do not show significant antiviral effect in preclinical models after a delayed administration post-infection (therapeutic regimen). At the time of writing, the preliminary results for **JNJ-64281802** in a phase II (DHIM, prophylactic regimen) clinical trial look favorable and make it the most promising compound, followed by **EYU688**. These compounds inhibit the viral machinery by targeting, respectively, NS3-NS4B and NS4B, and they show cellular antiviral efficacy ranging from nanomolar to subnanomolar, unlike the majority of the compounds that did not demonstrate clinical antiviral efficacy. This emphasizes the importance of developing novel NS2B-NS3 inhibitors, of which only **doxycycline** is currently in clinical trials. However, its precise mechanism of action and clinical efficacy as a viral growth inhibitor have yet to be established and demand further investigation.

To date, only one competitive NS2B-NS3 protease inhibitor, **6-bromo-1,2-naphthalenedione**, has been reported with in vivo efficacy. However, it exhibits limited broad-spectrum orthoflaviviral efficacy, primarily demonstrating selectivity toward ZIKV. **SYC-1307**, an allosteric inhibitor, is the most promising NS2B-NS3 inhibitor, exhibiting high in vivo efficacy that correlates with high potency and efficacy in vitro. **Temoporfin** and **methylene blue** represent the most promising orthosteric non-competitive inhibitors, although their in vivo efficacy is limited compared with **SYC-1307**.

Among the competitive inhibitors with potent cellular efficacy, **Compound 71**, a non-polycationic NS2B-NS3 inhibitor, emerges as the leading preclinical candidate due to its high cellular antiviral efficacy, minimal cytotoxicity, and favorable in vitro pharmacokinetic parameters. These attributes position it as an ideal candidate for potential advancement to in vivo studies. The development of competitive NS2B-NS3 inhibitors faces several challenges, including the dynamic nature of the mechanism of action of NS2B-NS3 and the use of artificial constructs in the biochemical inhibition assays and in X-ray crystallography and other techniques for structure elucidation. Furthermore, the very shallow binding area, which exhibits a preference for binding cationic residues in the natural substrate, contributes to the difficulty in developing competitive inhibitors.

## Figures and Tables

**Table 2:** Orthosteric NS2B-NS3 inhibitors with in vivo efficacy.

Drug	Virus	NS2B-NS3 IC_50_ (μM)	Cellular EC_50_ (μM)	Peak Viremia Reduction or SR % Dosage (Infection Model)	Year ^[a]^	Ref.
**Temoporfin**	DENV2	1.1 ± 0.1	0.020	2.0-log 1.6 mg/kg, IP (ZIKV GZ01, Balb/c)	2017	[127]
	ZIKV		0.024			
	WNV		0.010			
**JMX0207**	DENV2	8.2	0.31	0.8-log 20 mg/kg, PO (ZIKV PRVABC59, A129)	2020	[128]
	ZIKV		0.3			
**Niclosamide**	ZIKV	12.3 ± 0.6	0.48 ± 0.06	33% 2–5 mg/kg, PO (DENV PL046, ICR suckling mice)	2018	[127,129,130,131]
	DENV	21.6	0.55 ± 0.05			
	WNV		0.54 ± 0.17			
**Methylene Blue**	DENV2	8.9	0.36	1.69-log 100 mg/kg, PO (ZIKV PRVABC59, A129 mice)	2020	[132]
	ZIKV		0.087–0.2			
**Erythrosin B**	DENV2	1.9	1.2	80% SR 200–400 mg/kg, PO (ZIKV PRVABC59, A129 mice)	2021	[133,134]
	ZIKV	1.7	0.62			
	WNV		0.66			
**6-Bromo-1,2-Naphthalenedione**	ZIKV	67.47	1	20–60% SR 100 μL (50 μg), IV (ZIKV MR766, AG129 mice)	2022	[135]
Nitazoxanide **(Prodrug)**	ZIKV	15.9 ± 0.9	1.48 ± 0.18	~90% SR 100 mg/kg, IG (JEV SH-JEV01, CK)	2017	[127,136,137,138,139]
Tizoxanide **(Active Metabolite)**	DENV2	0.1	n.a.	n.a.		

^[a]^ Year relative to the latest publication about the compound at the time of writing. n.a. = not available.

**Table 3:** Allosteric NS2B-NS3 inhibitors with in vivo efficacy.

Drug	Virus	NS2B-NS3 IC_50_ (μM)	Cellular EC_50_ (μM)	Peak Viremia Reduction or SR% Dosage (Infection Model)	Year	Ref.
**SYC-1307**	ZIKV	0.20 ± 0.01	0.3–0.6 ^[c]^	96–98% 15 mg/kg, IP (ZIKV FLR, HN16; C57BL/6)	2019	[140,141]
	DENV2	0.59 ± 0.02				
	DENV3	0.59 ± 0.06				
	WNV	0.78 ± 0.02				
**Compound 1**	ZIKV	158 ± 25	13.9 ± 0.4	100% SR 1 mg/kg, IP (ZIKV Uganda, ICR)	2022	[142]
**Novobiocin**	ZIKV	14.2 ± 1.1	42.63	100% SR 150 mg/kg, SC ^[a]^ (ZIKV PRVABC59, BALB/c ^[b]^)	2017	[143,144]
**NSC157058**	ZIKV	0.82	50	~10-fold 30 mg/kg, PO (ZIKV PA259459, SJL mice)	2017	[78]
	WNV					
	DENV2					

^[a]^ Higher dose led to lethargy and body weight loss. ^[b]^ Dexamethasone-immunosuppressed ^[c]^ EC68.

**Table 4:** Orthosteric competitive NS2B-NS3 inhibitors with relevant (<20 µM) antiviral cellular efficacy.

Drug	Virus	IC_50_ (μM)	EC_50_ (μM)	CC_50_ (μM)	Cell Line	Year	Ref.
**Compound 71**	DENV2	8.0	0.24	>100	Huh-7	2021	[61]
	WNV	23					
**NK-189**	DENV2	11	0.89	>50	Huh-7	2020	[59]
	WNV	13					
**Compound 11d**	DENV2	2.5	4.1	>50	Huh-7	2019	[145]
**Compound 104**	DENV2	0.18	3.4	>100	Huh-7	2015	[72]
	WNV	0.56	15.5	>100			
**Tolcapone**	DENV1	1.1			BHK-21	2016	[146]
	DENV2	0.98	2.0	29			
	DENV3	0.91					
	DENV4	0.64					
	WNV	0.70					
**Compound-C**	DENV1	4.1			BHK-21	2016	[146]
	DENV2	4.0	9.0	76			
	DENV3	2.9					
	DENV4	3.4					
	WNV	2.5					
**6-APNP**	DENV4 ZIKV	n.a. 1.5	7.1 0.69–5.0 ^[a]^	35	Huh-7	2021	[147]

^[a]^ Range depending on ZIKV strain. n.a. = not available.

## Data Availability

Not applicable.

## Abbreviations

ADE, antibody-dependent enhancement; DENV, dengue virus; DHIM, dengue human infection models; ER, endoplasmic reticulum; HCV, hepatitis C virus; IP, intraperitoneal; IV, intravenous; ISRCTN, international traditional medicine clinical trial registry; JEV, Japanese encephalitis virus; LLOD, lower limit of detection; NHPs, non-human primates; NS, non-structural (protein); PO, per os, oral administration; PR, prophylactic regimen; SC, subcutaneous, intradermal; SR, survival rate; TBEV, tick-borne encephalitis virus; TH, therapeutic regimen; WNV, West Nile virus; YFV, yellow fever virus; ZIKV, zika virus.
